# In Vitro Testing of Artificial Sweeteners with Apple and Chokeberry Fruit Juice Extracts for Intestinal Permeability and Glucose Uptake

**DOI:** 10.1021/acsomega.5c06005

**Published:** 2026-03-31

**Authors:** Magdalena Köpsel, Tina Kostka, Tuba Esatbeyoglu

**Affiliations:** † Department of Molecular Food Chemistry and Food Development, Institute of Food and One Health, 26555 Gottfried Wilhelm Leibniz Universität Hannover, Am Kleinen Felde 30, 30167 Hannover, Germany; ‡ Division of Food Chemistry and Toxicology, Department of Chemistry, RPTU Kaiserslautern-Landau, Erwin-Schrödinger-Strasse 52, 67663 Kaiserslautern, Germany

## Abstract

Higher incidences of sugar-induced obesity and diseases such as diabetes force the development of healthy soft drink alternatives. Fruit juices, rich in beneficial polyphenols and sweeteners instead of sugar, could be a suitable option. The aim of this study was to investigate the influence of the sweeteners saccharin, cyclamate, acesulfame K, aspartame, and sucralose individually and in combination with fruit juice extracts of apple and chokeberry on human intestinal cells. Therefore, a coculture of Caco-2 and HT29-MTX cells was differentiated to form the intestinal epithelium, whereby the mechanistic focus was on intestinal barrier integrity versus glucose transport. In the in vitro system, saccharin alone and in combination with apple extract significantly increased the permeability, while apple polyphenols increased the glucose uptake. This increase was independent of changes in the expression of glucose transports SGLT1, GLUT 1, and GLUT 2. In contrast, aspartame significantly reduced the TEER values, which was prevented by apple polyphenols, but did not affect the permeability or glucose uptake. Thus, sweeteners may influence different mechanisms in the human intestinal cells. In conclusion, polyphenol-rich extracts can reduce or strengthen the effects induced by sweeteners and are, therefore, a promising additive for healthier and sugar-free beverages.

## Graphical abstract

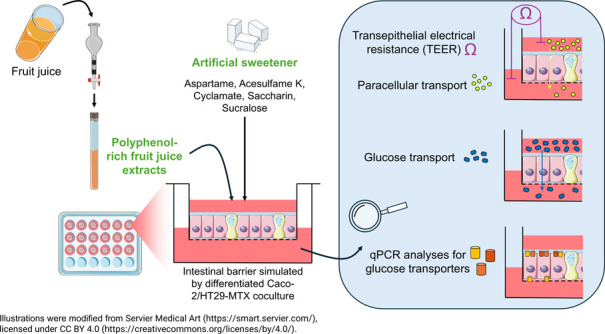

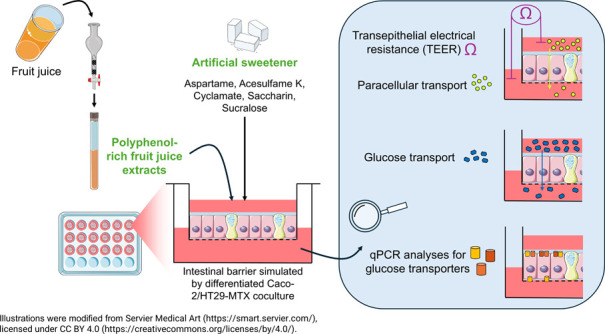

## 1 Introduction

One of the main challenges of today’s society is the increasing incidence of various metabolic diseases, such as type II diabetes, obesity, and cardiovascular disease.^1^ These diseases are closely linked to the consumption of sugar and sugar-sweetened beverages (SSB).^2–4^ While manufacturers add free sugars (i.e., monosaccharides and disaccharides), honey, syrup, and sugar-containing fruit juice concentrates to foods and beverages, the World Health Organization recommends reducing the intake of free sugars to less than 10% of total energy intake.^5^ To obtain sugar-free or healthier beverages, the use of artificial sweeteners as well as health-beneficial bioactive compounds or a combination of both could be promising. Especially the combination of both possibilities is a scientific gap, which should be characterized for its beneficial effects.

Fruit juice provides a significant amount of bioavailable micronutrients and beneficial plant bioactives, in similar amounts to whole fruits.^6^ In addition to carbohydrates (fructose, glucose, sorbitol, and sucrose), organic acids (ascorbic acid, citric acid, malic acid, and shikimic acid), amino acids (asparagine, proline, glycine, serine, methionine, and alanine), and phenolic compounds (catechin, chlorogenic acid, and rutin) are among the most important components of fruit juices. Especially the phenolic compounds are well-known for their antioxidant, anti-inflammatory, and cardioprotective potential.^7^ Therefore, the use of bioactive and sugar-free fruit juice extracts may be a suitable ingredient for more healthy SSB alternatives. Moreover, the absorption of sugar can be reduced by polyphenols. Previous studies already showed that fruit juice extracts significantly reduced the gene expression of glucose transporters (GLUT1 and GLUT2) in an in vitro intestinal epithelium model.^8^ Similar effects were seen in a human nutrition study. The consumption of a polyphenol-rich extract before meals significantly reduced the change in the plasma glucose level.^9^

Another opportunity is the replacement of sugar by alternative sweeteners for the production of new healthy beverages.^10^ In recent decades, the use of artificial sweeteners in food has increased significantly, forced by both consumers and food manufacturers.^11,12^ Artificial sweeteners are used to reduce calorie content and the risk of tooth decay while enhancing the flavor and taste of foods.^13^ These sweeteners are not nutritious and 30–13,000 times sweeter than sugar.^14^ People who are overweight or suffer from diabetes consume these substances instead of household sugar in order to control their carbohydrate intake, lower blood sugar levels, and also control their body weight.^15^ Imamura and colleagues confirmed the positive association of sugar-sweetened beverages with the occurrence of type 2 diabetes, while the association with artificially sweetened beverages or fruit juices was significant but less strong.^3^ In another study, the consumption of sweeteners has been associated with a lower risk of type 2 diabetes, weight gain, and obesity.^16,17^ However, other research suggests that sweetener consumption may increase the risk of diabetes, obesity, and even cancer.^18^ Thus, it is still disputed whether increased consumption of artificial sweeteners has a detrimental or beneficial effect in the treatment of obesity and diabetes.^19^ According to the study by Gougeon et al.,^20^ no significant effects on insulin levels, blood glucose concentrations, or blood lipid levels were found for the sweeteners saccharin cyclamate, aspartame, acesulfame K, and sucralose. Artificial sweeteners appear to be active in the gastrointestinal tract and affect the taste receptors of the gut, which could lead to increased insulin secretion, which in turn could affect weight, appetite, and blood glucose levels.^21^ Sweetener compounds behave very differently in terms of absorption in the gut, ranging from absorbable compounds to those that remain unchanged in the gut.^22,23^ Sucralose, aspartame, and saccharin have been shown to change the diversity of gut microbiota, leading to dysbiosis, which has been linked to metabolic diseases such as obesity and type 2 diabetes.^24,25^ Furthermore, a long-lasting change in the gut microbiota is associated with obesity and a “leaky gut” syndrome. The resulting disruption of the mucosal barrier and associated increased epithelial permeability allows the passage of lipopolysaccharides (LPS) from the gut microbiota, which in turn can lead to metabolic endotoxemia.^26,27^

Due to this current state of knowledge, it is hypothesized that the beneficial effects of phenolic compounds in combination with artificial sweeteners will reduce the glucose uptake and permeability in the gut. Especially, synergistic effects by combinating both of these factors could further strengthen human health. The aim of the study was therefore to investigate the influence of the sweeteners saccharin, cyclamate, acesulfame K, aspartame, and sucralose individually and in combination with the fruit juice extracts of chokeberry (*Aronia melanocarpa*) and apple (*Pyrus malus*) on the permeability of the intestinal barrier. Thus, for the first time, a mixture of different sweeteners with or without polyphenol-rich extracts were directly compared and analyzed in a more physiological intestinal coculture model. For the in vitro model of the intestinal barrier, a coculture of Caco-2 and HT29-MTX cells was differentiated to form the intestinal epithelium including mucus secretion. To investigate the effects on the permeability of the intestinal barrier, the transepithelial electrical resistance (TEER) of the intestinal membrane and the transport of the fluorescent marker molecule sodium fluorescein were determined. Furthermore, the actual glucose transport across the intestinal membrane as well as the transcriptional level of individual glucose transporter genes such as SGLT1, GLUT 1, GLUT 2, and DPP4 as well as the genes of the antioxidant enzymes SOD and CAT were analyzed.

## 2 Materials and Methods

### 2.1 Fruit Juices for the Extraction of Phenolic Compounds

Both of the fruit juices used (chokeberry and apple) are commercially available as direct juices. Chokeberry (*A. melanocarpa*) was obtained from Aronia Original Naturprodukte GmbH (Dresden, Germany), while the apple juice (*P. malus*) was provided by Obstkelterei van Nahmen GmbH & Co. KG (Hamminkeln, Germany).

### 2.2 Preparation of Fruit Juice Extracts by the XAD-7 Adsorption Column

Fruit juice extracts were prepared by extraction using an Amberlite XAD-7 (Sigma-Aldrich, Steinheim, Germany) adsorption column as described in Köpsel et al. and Kostka et al.^8,28^ While phenolic compounds bind to the XAD-7 resin, other compounds like minerals, organic acids, proteins, and carbohydrates were removed from the samples. In summary, for both juices, 0.5 L was applied onto the column (100 × 7 cm). After the washing step with water, the phenolic compounds were eluted with 19:1 (*v*/*v*) ethanol/acetic acid and freeze-dried to obtain a concentrated XAD-7 extract.

### 2.3 Separation of Individual Fractions from XAD-7 Fruit Juice Extracts

After the extraction of phenolic compounds, the XAD-7 extracts were fractionated into anthocyanins and copigments, defined as the residual phenolic compounds except anthocyanins. The fractionation was performed as described in Niesen et al.^29^ and Köpsel et al.^8^ using a Sartobind S IEX cellulose membrane (Sartorius, Göttingen, Germany). Briefly, 6 g of each extract was dissolved in 1 L 19:1 (*v*/*v*) methanol/acetic acid prior to application on the membrane. For elution of the copigment fraction, the membrane was washed with 19:1 (*v*/*v*) methanol/acetic acid. Anthocyanins were specifically eluted using 1 L of 1:1 (*v*/*v*) NaCl (1 N) and methanol. To remove NaCl from the anthocyanin solution, a second extraction by the XAD-7 adsorption column was performed.^30^

### 2.4 Cell Line Cultivation

For the following in vitro studies, the human colorectal adenocarcinoma epithelial cell line Caco-2 (German Collection of Microorganisms and Cell Cultures GmbH, Braunschweig, Germany) and the human colon adenocarcinoma goblet cell line HT29-MTX (European Collection of Authenticated Cell Cultures, Porton Down, UK) were used. Both cell lines were cultured using Dulbecco’s modified Eagle’s medium (DMEM, high glucose) supplemented with 10% (*v*/*v*) fetal bovine serum (FBS), 100 U/mL penicillin, 100 μg/mL streptomycin, and 1% nonessential amino acids (all from PAN Biotech, Aidenbach, Germany). All cell lines were kept at 37 °C in a humidified atmosphere containing 5% CO_2_. The cell passages ranged from 10 to 26, while the medium was changed every 2–3 days. For the cocultivation of Caco-2 with HT29-MTX cells, a ratio of 7:3 (Caco-2: HT29-MTX) was used.^31^

### 2.5 Testing for Cytotoxicity by Resazurin Reduction Assay

The viability of cell cultures was monitored by mitochondrial metabolic reduction of the nonfluorescent resazurin forming the fluorescent resorufin. The cytotoxic potential was analyzed for the artificial sweeteners acesulfame K, aspartame, saccharin, sodium cyclamate, and sucralose (all >98% purity, Sigma-Aldrich). For each well of a 96-well plate, 1.75 × 10^3^ Caco-2 and 0.75 × 10^3^ HT29-MTX cells (in total 2.5 × 10^3^ cells per well) were seeded in coculture and differentiated over 14 days by regular change of the medium. The differentiated cells were treated with artificial sweeteners, which were dissolved in Hanks’ Balanced Salt Solution (HBSS; PAN Biotech), for 24 h. HBSS was used as a negative control, while 1% (*v*/*v*) Triton-X (Carl Roth, Karlsruhe, Germany) served as a positive control. After treatment, the media was removed, followed by a 2 h incubation with 100 μL/well of 10% (*v*/*v*) resazurin solution. Finally, the fluorescence was measured using an Infinite M200 UV–vis spectrophotometer (Tecan, Crailsheim, Germany) with 560 nm as the excitation wavelength and 690 nm as the emission wavelengths. Cell-free wells containing the resazurin solution were used as blank samples. The cell viability was calculated by normalization to the values of the HBSS solvent control. For the following cell culture experiments, noncytotoxic concentrations of the sweeteners were used. Due to comparative mechanistic purposes, for all sweeteners, the same concentrations were tested, while regulatory limits and acceptable daily intakes were discussed below.

### 2.6 Co-culturing of Caco-2 and HT29-MTX Cells in TC-Inserts

To simulate the intestinal epithelium, Caco-2 and HT29-MTX cells were cocultured on TC-Inserts (0.3 cm^2^ growth area insert, 1 × 10^8^ pore density, 0.4 μm pore diameter; Sarstedt, Nümbrecht, Germany) placed onto a 24-well plate. In total, 5 × 10^4^ cells (3.5 × 10^4^ Caco-2 cells and 1.5 × 10^4^ HT29-MTX cells) were seeded in 200 μL of medium per insert (apical side). The wells below (basolateral side) contained 800 μL medium per well. The cells differentiated over 21 days, while the differentiation and increase in barrier function was regularly monitored by measuring the transepithelial electrical resistance (TEER). For the transport experiments, only inserts with constant and high TEER values were used.

### 2.7 TEER Measurements

The electrical resistance of cocultured cells in inserts was checked using a STX1 electrode with a Millicell ERS-2 device (Millipore, Bedford, MA, USA). For each insert, the cell-free TEER (prior to the cell seeding) served as a reference and was included in the calculation of the TEER as Ω cm^2^. For 24-well plates, constant values above 300 Ω cm^2^ indicate well-differentiated epithelial cells, which could be used for the below-mentioned transport experiments.

### 2.8 Epithelial Transport of Sodium Fluorescein and Glucose

By using the differentiated intestinal cell model, the influence of artificial sweeteners on the barrier function was analyzed. The fluorescent marker molecule sodium fluorescein is transported by the paracellular pathway from the apical to the basolateral side of the epithelium.^32^ Thus, the substance is an ideal marker for testing the permeability, which may be affected by the sweeteners. Furthermore, the glucose uptake by specific epithelial transporters was analyzed in the same setup. While previous studies showed a polyphenol-dependent inhibition of 1–10 mM glucose uptake in Caco-2 cells after several minutes, a concentration of 10 mM glucose was used.^33,34^ In a recent publication, the short-term effects after 4 h of treatment using sweeteners and polyphenols were analyzed. The 4 h treatment also enables the use of HBSS as a treatment solution independent of FBS, glucose, or phenol red, which would interfere with the photometric analyses. Only the TEER measurements were continued for up to 24 h to detect any recovery of the simulated epithelium. As described in Section 2.6, the differentiated intestinal epithelia were cultured for 21 days. At the beginning of cell treatment, the insets were washed using PBS, followed by treatment with 500 μL of 2 mM artificial sweeteners in combination with 10 mM glucose and 5 μM of sodium fluorescein dissolved in HBSS. As part of the combination studies, 1000 μg/mL of fruit juice extract was included in the treatment solutions. The cells were treated for 4 h, while 200 μL samples of both compartments (insert and well) were collected after 2 h and 4 h. These samples were stored at −20 °C until being analyzed for glucose and sodium fluorescein transport across the epithelium. To control the integrity of the epithelium and exclude cytotoxic effects by the used substances, the TEER was measured at several time points (1–24 h after start of treatment). Therefore, the treatment solution was changed to a regular cell culture medium after 4 h of treatment. Capric acid (C10; Carl Roth) is well-known for its permeability-increasing effects and was used with a concentration of 10 mmol/L as a positive control. Further controls in the experiment were a cell-free insert as well as a cell-containing insert treated with the solvents of the test compounds.

### 2.9 Quantification of Permeability by Sodium Fluorescein

Sodium fluorescein can be measured by a microplate reader (excitation, 460 nm; emission, 515 nm). In brief, a sodium fluorescein stock solution (50 mg/mL in HBSS) was prepared by dissolution. Subsequently, the stock solution was diluted with HBSS to obtain a working solution of 5 μM sodium fluorescein, which was stored protected from light at −20 °C until further use. For calibration, sodium fluorescein standards in the range of 0.01–0.5 μmol/L were diluted with HBSS. Finally, the samples from the apical sides of the transport experiment were diluted 1:20 (*v*/*v*) with HBSS. About 65 μL of the samples, sodium fluorescein standards, and HBSS (served as blank) were added in triplicate to a 96-well plate (Sarstedt). The fluorescence was measured (excitation, 460 nm; emission, 515 nm). The results were calculated using the sodium fluorescein standard curve and expressed as the percentage sodium fluorescein content in the apical compartment relative to the content at time 0 h of the simulated intestinal barrier. The apparent permeability coefficient (Papp) was calculated as described by Koepsel and colleagues.^8^

### 2.10 Quantification of Epithelial Glucose Uptake

The glucose concentration of the basolateral compartment was analyzed according to Köpsel et al.^8^ using a d-glucose assay kit (glucose oxidase/peroxidase; GOPOD; Elabscience, Houston, USA). The colorimetric reaction was measured at 505 nm with an Infinite M200 UV–vis spectrophotometer (Tecan). The concentration was calculated in reference to the starting concentration of the insert at *t* = 0 h.

### 2.11 Gene Expression Analyses by qPCR

The mRNA expression levels of several glucose transporters and antioxidant proteins of differentiated treated cells were analyzed as described in Köpsel and colleagues.^8^ Briefly, 4.2 × 10^4^ Caco-2 cells with 1.8 × 10^4^ HT29-MTX cells were seeded in 6-well plates and differentiated for 14 days. The cells were treated with 2 mM artificial sweeteners for 4 h. Afterward, the cells were harvested by trypsinization and washing steps. The samples were stored at −80 °C until extraction. The RNA extraction was performed using TRIzol (Thermo Fisher Scientific, Darmstadt, Germany) according to the manual. In the end, the integrity and purity of the extracted RNA was checked by agarose gel electrophoresis (2%) and UV absorbance (A260/A280 nm) using a Nanodrop One C (Thermo Fisher Scientific).

cDNA-synthesis was performed as described by Köpsel et al.^8^ At first, the linearity of the synthesis was verified by using different dilutions of a sample and a control. Afterward, the samples were diluted 1:30 with RNase-free water and treated with the DNase digest Mastermix to exclude DNA contaminations. The synthesis was performed using the M-MLV Reverse Transcriptase kit (Thermo Fisher Scientific). Samples without the addition of reverse transcriptase (-RT) were used as controls. All cDNA samples were stored at −20 °C.

For qPCR analyses, 2 μL of each cDNA sample was mixed together to be used as standards in a serial dilution. The samples were 1:2 diluted with RNase-free water, followed by mixing 2 μL of these diluted samples with 8 μL of a SYBR green-based Mastermix with individual primers of the target genes (details about the primers are described in Section 2.12). Each sample was analyzed in triplicate. To exclude the possibility of formed primer dimers or other contaminants, controls without cDNA were included for each primer pair. The qPCR was performed using the following protocol with a QuantStudio3 Real-Time PCR Thermocycler (Thermo Fisher Scientific): 2 min at 50 °C and 10 min at 95 °C, followed by 40 cycles of 15 s at 95 °C and 60 s at 60 °C, and finally including the melting curve analyses using 15 s at 95 °C, 60 s at 60 °C, and a temperature increase of 0.15 °C per sec up to 95 °C.

### 2.12 Primer-Establishment

For primer establishment, RT-qPCR was performed with the designed primers and the corresponding cDNA. Agarose gel electrophoresis was then used to determine the size of the resulting product.

The separation was performed in 2% agarose gels. The RNA samples were thawed, diluted 1:4 with RNase-free water, and mixed with 1 μL of loading buffer (Thermo Fisher Scientific, Darmstadt, Germany). To estimate the size of the bands, a size standard (1 kbp DNA ladder) was applied. The electrical voltage during gel electrophoresis was 120 mV. The DNA bands were detected under UV light using the Imager iBright 1500 documentation system (Thermo Fisher Scientific, Darmstadt, Germany). To assess the efficiency and quality of the primers, the cDNA was prepared in a standard dilution. A triplet of each cDNA strand was applied. In order to assess the efficiency, the mean value was calculated from the Ct values obtained. The mean values of the Ct values were plotted graphically against the logarithm of the concentration of the cDNA. This resulted in a straight line, from which the slope was determined. The coefficient of determination should assume values between 0.98 and 1. The efficiency was calculated by a Thermocyler (QuantStudio3 Real-Time PCR System) and converted into the percentage efficiency. The formation of secondary products was excluded by analyzing the melting curves. Table S1 shows the primer pairs used in this study and the efficiencies in each case.

### 2.13 Statistical Analysis

All data were calculated as the mean ± standard deviation (SD) for at least three independent experiments. The evaluation was performed using the software Prism (version 10.1.1; GraphPad, La Jolla, CA, USA). At first, the data were analyzed for normal distribution using the Shapiro–Wilk test. In case of normality, the samples were compared by one-way ANOVA including Tukey’s multiple comparison test. Alternatively, in the case of missing normal distribution, the samples were statistically compared by the Kruskal–Wallis test with Dunn’s multiple comparison test. Statistically significant differences were given at *p* < 0.05.

## 3 Results

### 3.1 Cytotoxicity

The artificial sweeteners used in soft drinks and multifruit juices can damage cultured cells if they are applied undiluted or too highly concentrated. The cytotoxic potential of the artificial sweeteners acesulfame K, aspartame, cyclamate, saccharin, and sucralose on the Caco-2 and HT29-MTX cells in coculture was first investigated, with a view to subsequent in vitro experiments in which various artificial sweeteners were used as test substances, using the resazurin assay. Concentrations were selected that had already been described in the literature using Caco-2 and HT29 MTX cells and at which an effect on the artificial sweeteners had already been observed.^27,35,36^ The results of the cytotoxicity tests are shown in Figure 1. It can be seen that the sweeteners acesulfame K, aspartame, cyclamate, and sucralose have no cytotoxic effects on Caco-2 and HT29-MTX cells in the coculture at all concentrations. Consequently, no EC_80_ value could be determined for these sweeteners, as none of the concentrations tested fell below a cell viability of 80%. The sweetener saccharin also showed no cytotoxic effect at concentrations of 5 and 10 mM. However, a significant decrease in relative cell viability to 10% of the control was observed when the cells were treated with saccharin at a concentration of 20 mM. As cell viability fell below 80% at this concentration, an EC_80_ value of 12.8 mM was determined. It was concluded that the use of the artificial sweeteners acesulfame K, aspartame, cyclamate, and sucralose up to concentrations of 20 mM and the sweetener saccharin up to concentrations of 12 mM did not impair the cell viability of Caco-2 and HT29-MTX cells in coculture. According to pre-experiments, the sweeteners saccharin, aspartame, and sucralose showed the most promising effects and were analyzed in the following assays. Therefore, noncytotoxic concentrations of 2 mM were used.

**Figure 1:**
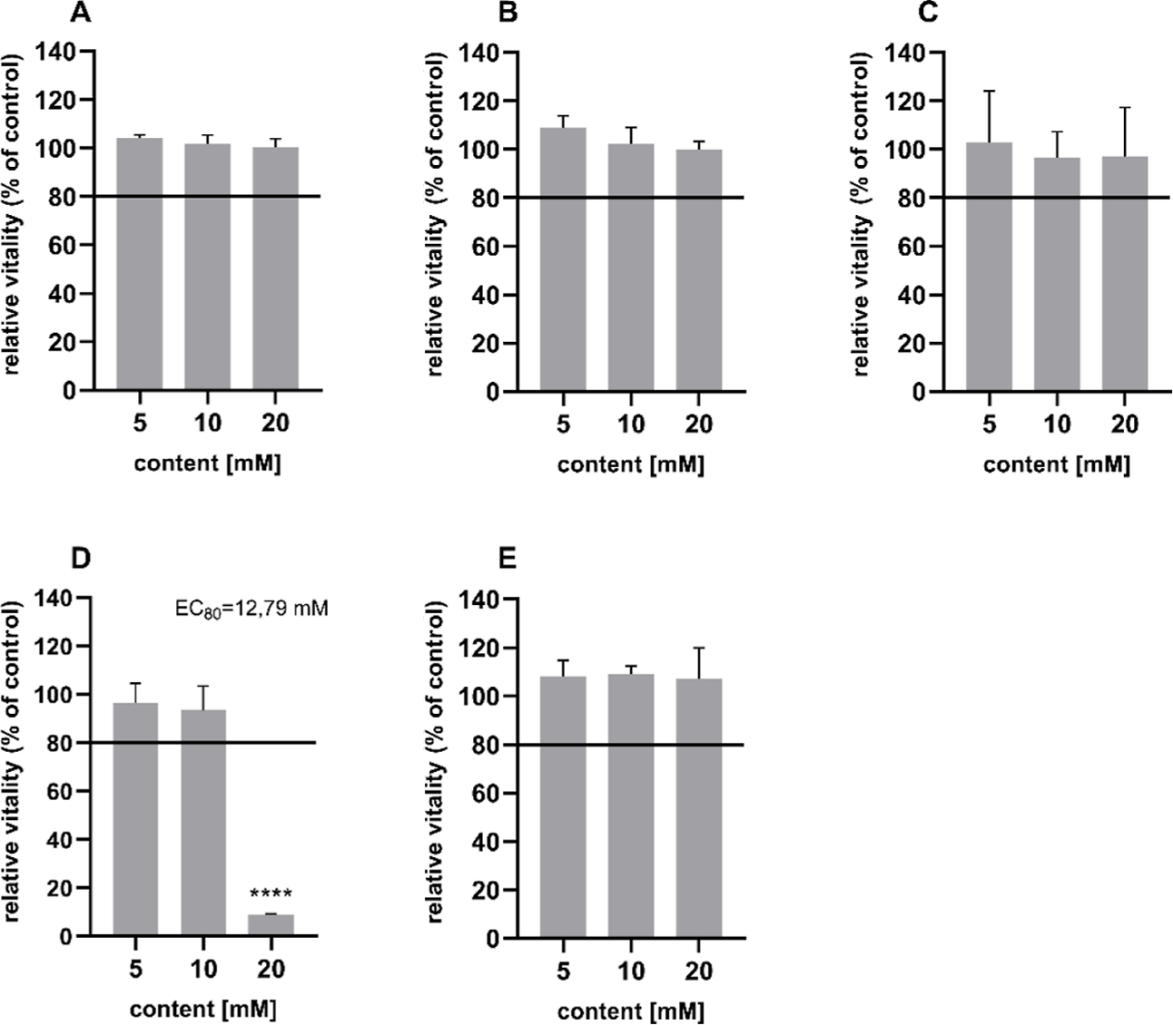
Cytotoxic effects after 24 h of treatment with the artificial sweeteners acesulfame K (A), aspartame (B), cyclamate (C), saccharin (D), and sucralose (E) on the cell viability of Caco-2 cells and HT29-MTX cells in coculture in the resazurin assay. Indicated in % of the viability of the untreated control (DMEM medium). *n* = 3 biological replicates. Mean ± SD; Shapiro–Wilk test followed by one-way ANOVA with Dunnett’s multiple comparison test; ****p < 0.0001. The included line at 80% viability highlights the calculated EC80 value.

### 3.2 Transepithelial Electrical Resistance (TEER)

To investigate the permeability of the intestinal barrier during the treatment of Caco-2 and HT29-MTX cells, TEER values were measured during the treatment of the cells, 6 h after the start of treatment (4 h treatment + 2 h recovery) and over a period of up to 24 h after the start of treatment (4 h treatment +20 h recovery), in order to observe possible cell regeneration. The most effective changes in the TEER values were seen after treatment. Therefore, the results of 6 and 24 h were visualized and discussed below.

As shown in Figure 2A, the TEER values decreased to 40–60% at 6 h after treatment with the respective artificial sweeteners compared to the initial value. The TEER value of the control (HBSS) remained at a similar level to the initial TEER value during and after the treatment of the cells. In comparison to the control, as well as in comparison to each other of the different sweeteners, no significant decreases in the TEER value can be recognized, except of aspartame. When the cells were treated with 2 mM aspartame, the strongest decrease in the TEER values in relation to the initial value was observed, while this decrease was significant after 4 h of treatment and 20 h of recovery.

**Figure 2:**
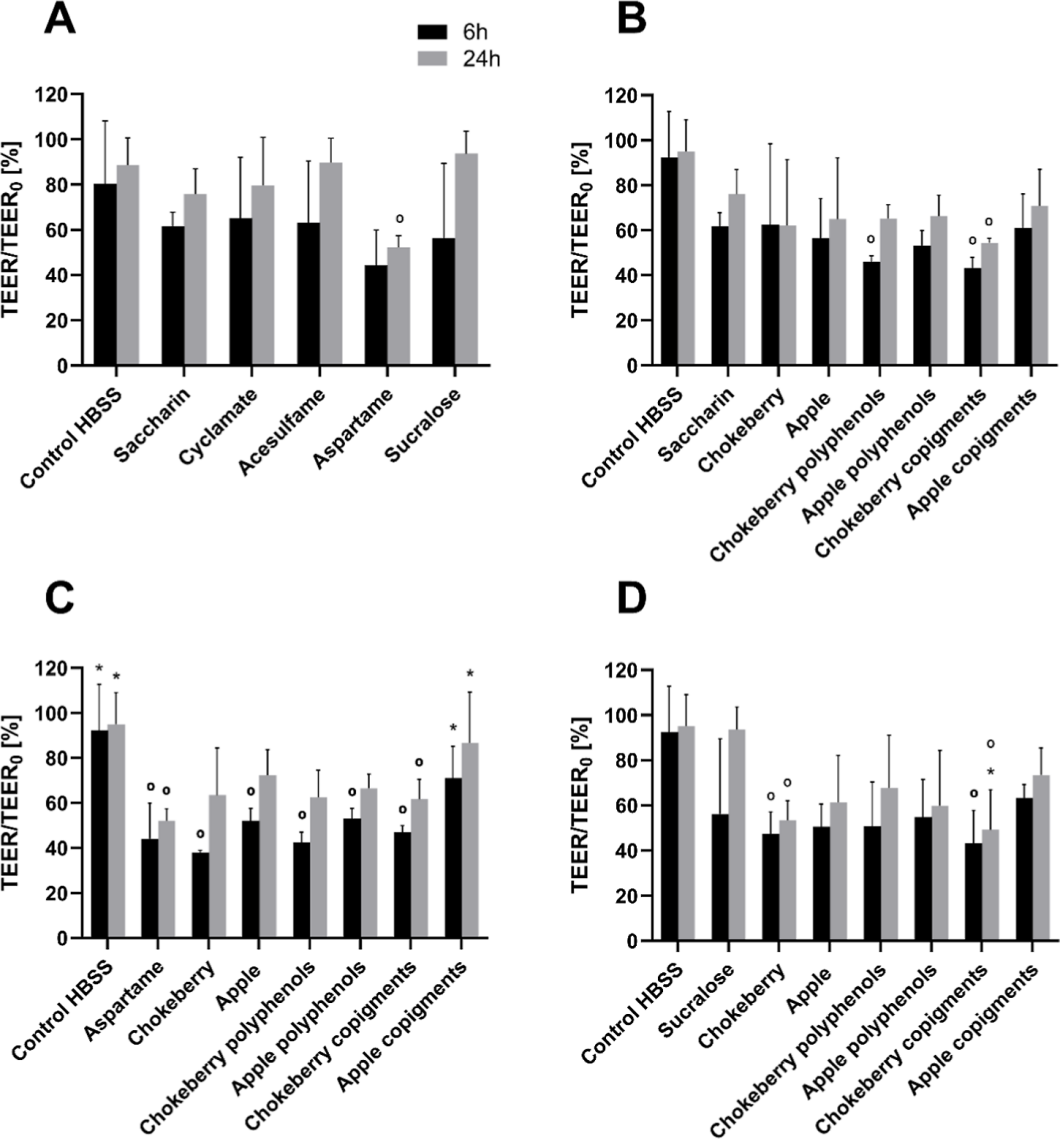
Percentage change in TEER values after 6 h (4 h treatment + 2 h recovery) and 24 h (4 h treatment + 20 h recovery) normalized to initial (0 h) TEER values when the simulated intestinal barrier was treated with artificial sweeteners with or without XAD-7 extracts in coculture of Caco-2 and HT29-MTX cells (2 mM artificial sweeteners +1000 μg/mL XAD-7 extract or polyphenol/copigments fraction + 5 μM sodium fluorescein). A: treated with the artificial sweeteners saccharin, aspartame, cyclamate, acesulfame and sucralose. B: treated with saccharin alone and in combination with XAD-7 extracts and polyphenols/copigment fractions. C: treated with aspartame alone and in combination with XAD-7 extracts and polyphenols/copigment fractions. D: treated with sucralose alone and in combination with XAD 7 extracts and polyphenols/copigment fractions. *p < 0.05 compared to the corresponding sweetener; °*p* < 0.05 compared to the HBSS control; *n* = 3 biological replicates. Mean ± SD; Shapiro–Wilk test followed by one-way ANOVA with Dunnett’s multiple comparison test.

When the cells were treated with the sweetener saccharin in combination with the fruit juice extracts and their fractions, a decrease in the TEER value after 6 h can be seen. While saccharin nonsignificantly reduced the TEER to 60%, the combination with fruit extracts of chokeberry polyphenols and chokeberry copigments induced a significant reduction in TEER value compared to the untreated control. Even after 20 h of recovery, saccharin with the chokeberry copigments led to a significant reduction in TEER. The TEER values of the apple extract and its fractions were comparable to and similar to those of the saccharin treatment.

The combined treatment with fruit extracts and aspartame showed comparable and significant results after 6 h except for the apple copigments (Figure 2C). Compared to the untreated control, the TEER values decreased to 40–60%. Aspartame alone reduced the TEER likewise. The copigment fraction showed TEER values significantly higher to the aspartame-only treatment for both time points. After 24 h, the cells treated with aspartame and apple copigments reached TEER values comparable to the untreated control. As shown in Figure 2C, the TEER values of all combined treatments regenerate after 24 h after treatment, reaching values of 60–80% compared to the initial value. Nevertheless, no significant differences can be seen here in comparison to the aspartame control and between the individual treatment solutions.

Similar to saccharin, there was no significant effect of sucralose alone. In combination with chokeberry extract as well as with the chokeberry copigments, sucralose significantly reduced the TEER values after 6 and 24 h. The results of all other combined treatments were nonsignificant. Interestingly, the combination of sucralose with chokeberry copigments significantly reduced the TEER values compared with the sucralose control after 24 h. In general, the treatment with the sweetener aspartame in combination with most of the fruit juice extracts showed the strongest decrease in TEER values after 6 h of treatment compared to the other sweeteners saccharin and sucralose.

#### 3.2.1 Transport of Sodium-Fluorescein

Different sweeteners in combination with different fractions of fruit juice extracts can have individual effects on the permeability of the intestinal barrier and the transport of the fluorescent marker sodium fluorescein. When the cells were treated with saccharin, the transport of sodium fluorescein increased significantly after 2 h. Treatment of the cells with saccharin after 4 h also slightly increased the transport of sodium fluorescein but not significantly compared to the control. All other artificial sweeteners showed no significant changes in the transport of sodium fluorescein compared to the control or to each other (Figure 3A). While saccharin increased the Papp value, the addition of the apple extract significantly increased the permeability after 6 h compared to the untreated control (Figure 3B). However, when the apple extract was separated into polyphenols and copigments, such an increase was not seen. In the treatment with saccharin and chokeberry extracts or fractions, no significant changes were detected. When the cells were treated with the sweeteners aspartame and sucralose in combination with the fruit juice extracts of chokeberry and apple as well as their individual fractions, no significantly higher or lower transport of sodium fluorescein was observed compared to the control (Figure 3C,D). Generally, it was observed that the treatment of the cells with the sweetener saccharin alone or in combination with the apple fruit juice extract but not its fractions resulted in a higher transport of sodium fluorescein compared to the control.

**Figure 3:**
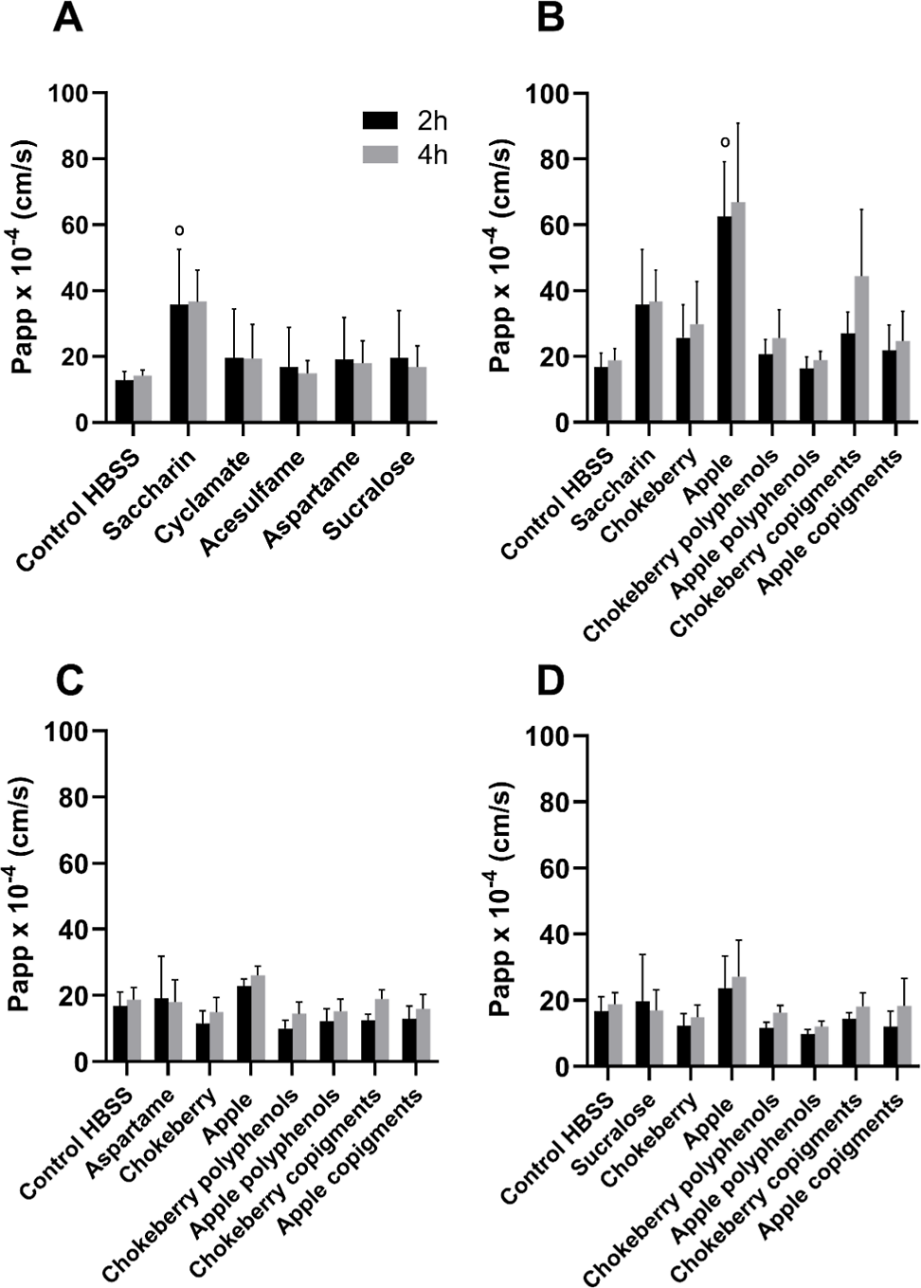
Effects of different artificial sweeteners in combination with XAD-7 extracts on sodium fluorescein transport (2 mM artificial sweeteners + 1000 μg/mL XAD-7 extract + 5 μM sodium fluorescein). A: treated with the artificial sweeteners saccharin, cyclamate, acesulfame, aspartame, and sucralose. B: treated with saccharin alone or in combination with XAD-7 extracts and polyphenols/copigment fractions. C: treated with aspartame alone or in combination with XAD-7 extracts and polyphenols/copigment fractions. D: treated with sucralose alone or in combination with XAD-7 extracts and polyphenols/copigment fractions. Mean ± SD (*n* = 3); Shapiro–Wilk test followed by one-way ANOVA with Dunnett’s multiple comparison test. °*p* < 0.05 compared to the HBSS control.

#### 3.2.2 Glucose Colorimetric Assay

The glucose oxidase/peroxidase (GOPOD) assay was used to measure the glucose content in the basolateral compartment after treatment of the cells with the different sweeteners in combination with the fruit juice extracts of apple and chokeberry as well as their individual fractions. The aim was to determine whether any of the sweeteners in combination with the fruit juice extracts had an effect on the transport or uptake of glucose into the cells. In Figure 4A, the results show that no significant changes in glucose transport were detectable after 4 h treatment of the cells with sweeteners. The glucose content was below 1 mmol/L for the control as well as for each of the sweeteners.

**Figure 4:**
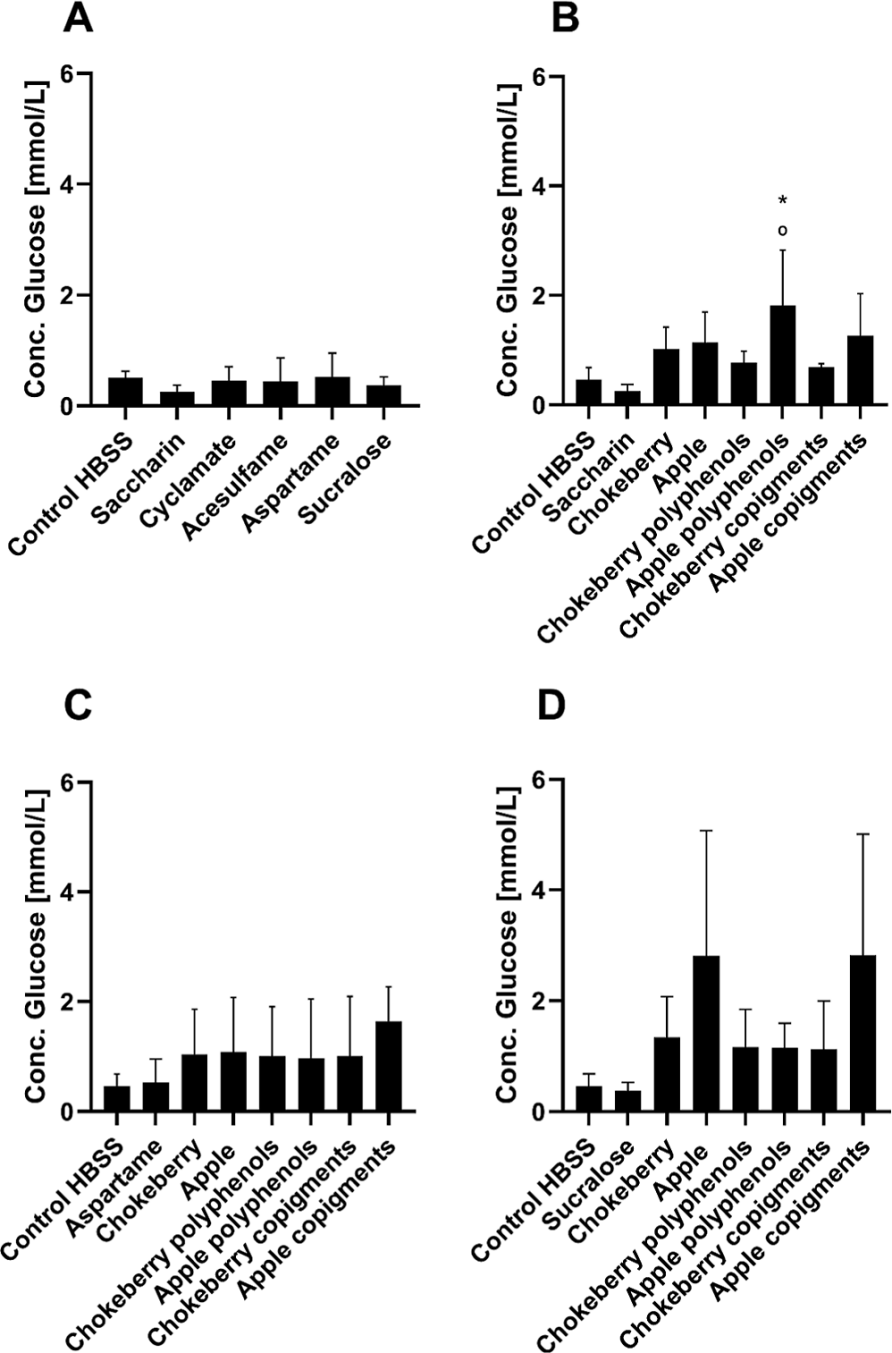
Effect of sweeteners with or without fruit extracts on the glucose transport under the simulated intestinal barrier in coculture of Caco-2 and HT29-MTX cells (2 mM artificial sweeteners + 1000 μg/mL XAD-7 extract and polyphenols/copigment fractions + 10 mM glucose). A: Comparison of the effect of the artificial sweeteners saccharin, cyclamate, acesulfame, aspartame, and sucralose on glucose transport. B: Comparison of the effect of saccharin in combination with XAD-7 extracts and polyphenols/copigment fractions on glucose transport. C: Comparison of the effect of aspartame in combination with XAD-7 extracts and polyphenols/copigment fractions on glucose transport. D: Comparison of the effect of sucralose in combination with XAD-7 extracts and polyphenols/copigment fractions on glucose transport. Mean ± SD (*n* = 3); Shapiro–Wilk test followed by one-way ANOVA with Dunnett’s multiple comparison test; *p < 0.05 compared to the corresponding sweetener; °*p* < 0.05 compared to the HBSS control.

Figure 4B shows that treatment with saccharin in combination with the apple polyphenol fraction significantly increases the glucose concentration in the basolateral compartment after a 4 h period. Such an increase was significant compared to the HBSS and saccharin control. The level was 4-fold higher compared to the untreated control and 7-fold higher compared to saccharin control. In comparison, the other apple extracts showed no significant effects. When cells were treated with the sweetener aspartame in combination with fruit juice extracts, none of the tested treatment solutions showed significant differences in comparison to aspartame or to each other (Figure 4C). While sucralose alone had no effect, Figure 4D shows that the fruit juice extract of the apple and the apple copigment fraction have a tendency to increase glucose transport into the basolateral compartment. These results were nonsignificant due to high deviation of the results. The treatments with sucralose and the other fruit juice extracts showed no effects in glucose transport. Overall, it can be seen that the results for the apple extracts and fractions might be able to increase the glucose transport especially in combination with saccharin.

### 3.3 Effects of Sweetener on the Transcription of GLUT1, GLUT2, SGLT1, DPP4, CAT, and SOD mRNA in Cocultures of Caco-2 and HT29-MTX Cells

Quantitative real-time PCR was used to determine the effects of the various sweeteners on the transcription of the glucose transporters GLUT1 and GLUT2 as well as the mRNA of the DPP4 and SGLT1 signaling pathways, which also play an important role in glucose uptake. Furthermore, the transcription of CAT and SOD, which belong to the antioxidant enzymes, was determined, whereby the effects of the polyphenol extracts and fractions were expected. In order to investigate the physiological intestinal retention of the different sweeteners, the cells were treated with the sweeteners for 4 h.

As can be seen in Figure 5 A–D, there was no significant reduction or increase in the expression of the glucose transporters GLUT1, GLUT2, SGLT, and DPP4 after a treatment time of 4 h compared to that of the control. Likewise, there was no significant change in the stress-induced genes CAT and SOD compared to the control or among the sweeteners (Figure 5 E,F).

**Figure 5:**
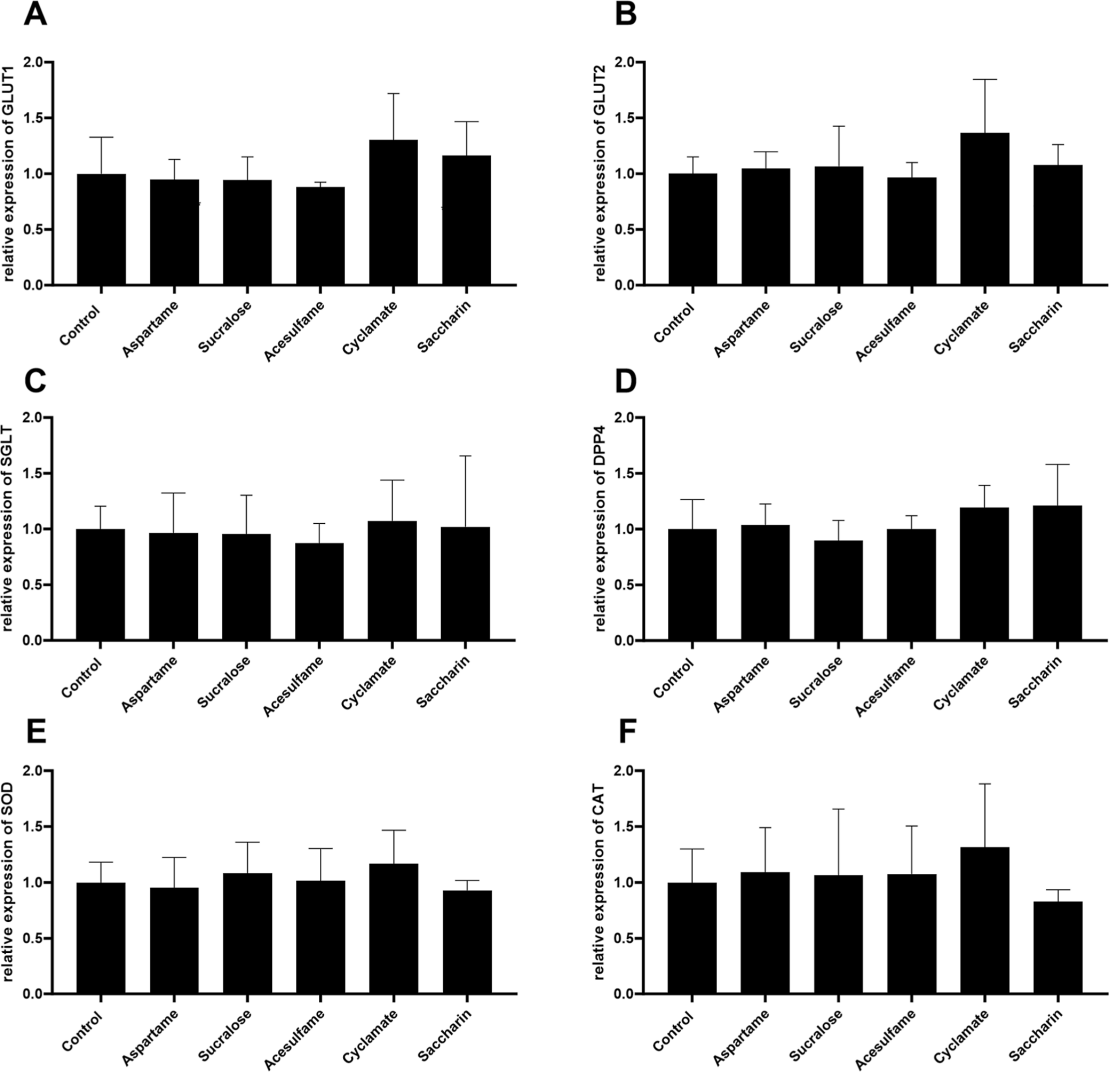
Relative mRNA expression values of GLUT1 (A), GLUT2 (B), SGLT (C), DPP4 (D), CAT (E), and SOD (F) normalized to the expression of 18S. Simulated intestinal barrier of Caco-2 and HT29-MTX coculture was treated for 4 h with artificial sweeteners aspartame, sucralose, acesulfame K, cyclamate, and saccharin (2 mM). MW ± SD (*n* = 3); Shapiro–Wilk test followed by one-way ANOVA with Dunnett’s multiple comparison test; nonsignificant compared to control.

## 4 Discussion

The aim of the study was to evaluate the role of the artificial sweeteners saccharin, aspartame, sucralose, cyclamate, and acesulfame K individually and in combination with fruit juice extracts on intestinal permeability. As described in the literature, the consumption of 100% fruit juice is being discussed more intensively due to its high content of free sugars. However, fruit juice also provides a significant amount of bioavailable micronutrients and plant bioactives, in similar amounts to whole fruits.^6^ Thus, artificial sweeteners, which could be formulated in combination with extracts from fruit juices, could be alternatives to fruit juices suitable for people suffering from metabolic disorders such as type II diabetes or obesity. The treatment of the cells in coculture with fruit juice extracts in combination with artificial sweeteners was limited to the fruits of apple and chokeberry in this study. Both belong to the same plant family as the rose family (Rosaceae). Chokeberries have excellent antioxidant properties due to their high concentration of anthocyanins. The apple is a representative of the yellow fruits, so that differences can possibly be made here with regard to the anthocyanins, since they are in smaller amounts in the peel. For the artificial sweetener, a concentration of 2 mM was chosen due to the European regulation of a maximum quantity of 600 mg/L aspartame in aromatic beverages and fruit nectar (EU regulation 1333/2008). The focus was to identify compound-specific differences independent of the individual sweetness and acceptable daily intake (ADI) values. Nevertheless, these factors were reviewed at the end of the discussion.

The used coculture model simulates the intestinal epithelium and enables the evaluation of permeability and uptake. An important factor that should be reconsidered is the impact of the tested sweeteners on the microbiome of the colon. It was shown that acesulfame K, sucralose, and saccharin led to dysbiosis, while acesulfame K further induced weight gain in treated mice and sucralose and saccharin increased the expression of pro-inflammatory genes.^22,24,37,38^ While the sweeteners affect the microbiome, these changes in composition can further change the metabolism and effects of the sweeteners. The regular consumption of sucralose significantly changed the found metabolites in the colon, e.g., tryptophan metabolism,^38^ which all can positively or negatively influence the intestinal permeability. Thus, the results of the recent study focus on in vitro experiments for permeability and glucose uptake, whereas microbial changes could be an additional important factor to reconsider.

### 4.1 TEER

TEER values were measured during and after incubation with sweeteners and fruit juice extracts to investigate the influence on the intestinal permeability. The tested artificial sweeteners saccharin, aspartame, sucralose, acesulfame, and cyclamate do not appear to have a significant influence on the TEER values of the coculture, except for aspartame after 24 h. Thus, aspartame showed the most effective increase in permeability compared to those of the other tested sweeteners. An increase in permeability by aspartame but not by sucralose was already shown in Caco-2 cells. The authors were able to show that aspartame, but not sucralose induce reactive oxygen species (ROS) formation, which correlates to the higher permeability.^39^ The noneffective treatment with most of the sweeteners is also reflected in the studies by ref 27. There, the influence of the five sweeteners was also investigated but only on Caco-2 monolayers. Thus, the impact of mucin secretion by goblet cells was not integrated into the model. There, only significant differences were noticeable in the treatment of the cells with saccharin, but the treatment time there was only 3.5 h instead of 4 h as in our study and the concentration of the sweeteners with a total of 10 mM was much higher than the concentration of 2 mM tested in the present study.^27^ Similar studies were performed by treating Caco-2 monolayers with 0.1 mM of sweeteners for 24 h, followed by permeability measurements.^39^ The longer incubation time induced a significant increase in permeability by aspartame and sucralose, while these effects were affected by the induction of sweet taste receptor T1R3. It was shown that T1R3 led to higher claudin-15 expression, which is involved in pore formation, and lower claudin-3 expression, responsible for tight junction sealing.^39^ The activation of T1R3 by artificial sweeteners was also shown in an in vivo study feeding mice with 2 mM sucralose. T1R3 induced higher SGLT1 expression, which was not detectable in T1R3–/– knockout mice.^40^ Therefore, the receptor T1R3 seems to be mainly involved in intestinal barrier function and SGLT1 expression and may induce lower TEER values in the recent study. For verification, more studies focusing on the correlation of sweeteners T1R3 and tight junction are needed.

Due to the fact that coculture from Caco-2 and HT29-MTX cells is used in the investigations, it can be assumed that the effects in relation to the TEER values of Santos et al.^27^ and Shil et al. 2020 ^39^ are not quite as strong, as a probably more stable monolayer is formed in coculture, as well as the formation of a protective mucus layer by the HT29-MTX cells. Xie et al.^36^ also showed that the Papp index, which was calculated on the basis of the TEER values in a coculture with Caco-2 and NCl-H716 cells after 30, 60, and 90 min, is higher when treated with glucose in combination with sucralose than in the pure Caco-2 monoculture. Santos et al.^27^ also showed that the effects of saccharin strongly depended on the combination of used concentration and incubation time. For instance, there was no difference in the TEER values when treating the cells with 3 or 10 mM saccharin. Even 1 mM increased the permeability significantly, but this process needed more time compared to the higher concentrations.^27^ The most reducing and significant effects were seen for 2 mM aspartame, and the TEER value remained below 60%, even after 20 h of regeneration. Interestingly, such a decrease of the TEER value by aspartame was similar for all polyphenol extracts but suppressed by cotreatment with apple copigments. Apple extract and apple polyphenols showed no significant difference from the aspartame control, while the copigments showed the strongest effect, comparable to the untreated control. Chokeberry extracts, as well as their fractions, did not influence the effect of aspartame. Therefore, anthocyanins may not be able to increase the intestinal barrier function, as already discussed in Köpsel et al.^8^ It was hypothesized that anthocyanins can prevent an increase in permeability by their antioxidant potential in ROS scavenging. On the other hand, anthocyanins like pelargonidin can rearrange the actin filaments, while tight junctions were delocalized in treated Caco-2 cells.^41^ However, the chokeberry copigments are free of anthocyanins but still led to low TEER values in combination with saccharin and sucralose. Other studies showed an increase in the intestinal barrier function by anthocyanins and other flavonoids. A moderate increase in barrier function (TEER values) and decrease in permeability flux by flavonoids in normal intestinal epithelial layers has already been established by the studies of Rowland et al.^42^ and Mercado et al.^43^ There is also evidence that treatment with flavonoids promotes the formation of tight junctions in High Glucose-Caco-2 cells. Of the flavonoids tested in the study by Carrasco-Pozo et al.,^44^ quercetin was the most effective, followed by naringenin and morin, in reducing oxidative stress and mitochondrial membrane depolarization. Similarly, polyphenols such as curcumin derivatives, quercetin and theaflavin have been reported to promote the restoration of transmembrane proteins (claudin family protein, occludin) and peripheral membrane proteins (zonula occludens) to improve the integrity of the mucosal barrier in normal cells.^44,45^ In contrast, the treatments with saccharin or aspartame in combination with the polyphenol fraction of chokeberry showed a relatively strong decrease after 6 h of treatment. It can therefore be concluded that the influence of the different fruit juice extracts is not necessarily due to their antioxidant effect, as chokeberry has a higher antioxidant potential than apple.^8^

Finally, the investigations in this study showed that the apple copigment fraction in combination with the sweetener aspartame can prevent or attenuate an aspartame-induced increase in intestinal permeability and that these extracts could be used, for example, in the treatment and prevention of chronic intestinal diseases. On the other hand, chokeberry extracts appear to increase intestinal permeability, which may be used, for example, to increase the absorption of substances via the intestinal membrane, such as medication. It would be interesting to analyze such effects on the efficient drug uptake by more complex or even animal models. In summary, yellow fruit extracts and not anthocyanin-rich red fruit extracts seem to be more suitable for combinations with sweeteners by reduction of the permeability increase.

### 4.2 Sodium-Fluorescein

To assess the actual transport of molecules across the intestinal membrane during or after the treatment of sweeteners in combination with fruit juice extracts, the transport of the fluorescent dye sodium fluorescein across the intestinal membrane was investigated.

In our study, the transport of sodium fluorescein was visualized in the form of a Papp index, which includes the initial concentration, the area of the membrane, and the time of treatment. A significantly higher transport of sodium-fluorescein was observed during treatment with the sweetener saccharin. Treatment of the cells with saccharin and the fruit juice extract of apple also showed a significantly higher permeability of sodium-fluorescein after 2 h compared with the saccharin and HBSS control. Therefore, both saccharin and the apple extract increased the Papp index. However, this increase by the apple extract was not detected in combination with aspartame and sucralose. All other treatments showed no significant differences in the transport of sodium fluorescein relative to that of the controls. In comparison of the apple extract with its fractions (apple polyphenols and apple copigments), the fraction-specific compounds are not responsible for the significantly higher Papp index in the apple extract. The apple polyphenol extract is rich in catechin, while the nonfractioned apple fruit juice extract possess significantly higher levels of chlorogenic acid (12.2 g/100 g extract), analyzed by Köpsel et al.^8^ The chokeberry copigment fraction is also rich in chlorogenic acid (6.60 g/100 g extract) and showed tendentially higher permeability in combination with saccharin.^8^

Independent of the composition of fruit juice extracts, saccharin increased the permeability but did not induce a significantly lower TEER value. Moreover, in the TEER measurements, aspartame induced a significant TEER reduction, but in the case of the transport of sodium-fluorescein, there was no effect by aspartame. This could possibly be an indication that the substance may not be transported through the intestinal membrane by increasing the permeability but that the transport could possibly also take place via other intracellular transport mechanisms. Similar independent results for TEER and Papp measurements were already seen for fruit juice extracts in the same coculture model.^8^ The missing correlation of these two end points could possibly also be an indication that the treatment time was not sufficient for adequate transport of the fluorescent dye through the simulated intestinal membrane. Furthermore, it is already known that TEER values depend on the electrode position, the temperature, as well as the ion flux.^46^ Polyphenols can affect the expression of tight junctions like claudin-4 and claudin-16, while these membrane proteins regulate the transport of sodium, magnesium, or calcium.^47,48^ Ion flux influences the TEER values but may not be relevant for the permeability assay using sodium fluorescein. Moreover, the physiological relevance of permeability and TEER measurements is different. If the ion flux especially for sodium is affected by the sweeteners and leads to the TEER changes, such an ion transport would influence the water reabsorption in the colon. It is known that chronic colon diseases with dysfunctional ion transport reduce colonic water absorption and higher risks of dehydration.^49^ In the case of higher permeability by paracellular transport or reduction of TJ, this leaky gut is sensitive for inflammatory responses induced by bacterial endotoxin uptake. Ideally, sugar alternatives should neither influence the ion flux nor increase the permeability.

By TEER evaluation, the combination of apple copigments with aspartame was identified as the most acceptable mixture of sweetener and bioactive compounds, which do not affect the intestinal barrier function. In the case of transport experiments, such a mixture does not influence the transport of sodium fluorescein and thus strengthens the suitability of aspartame and apple copigments as healthier alternatives for sugar in beverages.

### 4.3 GOPOD

In addition to the TEER measurements and the paracellular transport, the cellular transport of glucose was investigated. Glucose transport is an important end point especially for the development of sugar-free beverages, suitable for diabetes patients. The coculture was treated with artificial sweeteners with/without fruit juice extracts, while 10 mM glucose was added to the apical compartment. After the treatment period, the passage of glucose in the basolateral compartment was determined using the GOPOD assay. No significant differences in glucose transport across the intestinal membrane as a function of the different treatments are visible when the cells are treated with the individual sweeteners. Previous studies have also shown that glucose uptake in Caco-2 and RIE-1 cells was significantly increased at glucose concentrations of >25 mM and at incubation times >5 min^50^ Furthermore, it was investigated whether the artificial sweetener acesulfame K can increase carrier-mediated glucose uptake in a cell culture system. When exposed to 10 mM acesulfame K, carrier-mediated glucose uptake was increased in Caco-2 and RIE-1 cells at 25 and 50 mM glucose for 5 min of incubation. At lower glucose concentrations (<10 mM), there was no increase at any incubation duration. Similarly, there was no effect of acesulfame K on glucose uptake during the 1- and 10 min incubation periods. In IEC-6 cells, no effect of acesulfame K on glucose uptake was observed at any of the incubation durations tested.^50^ Even if there were no significant effects induced by the sweeteners in the recent study, some tendencies between the treatments of the sweeteners in combination with the individual fruit juice extracts and fractions can be recognized.

In general, the fruit juice extracts showed higher glucose levels in the basolateral compartment, which is in correlation with the lower TEER values. The reduction in TEER results indicated a higher permeability and, therefore, higher levels of measured glucose. According to this point, it should be highlighted that in the case of a higher permeability, the glucose may be transported by the paracellular pathway and not actively by the cells. This passive transport of glucose was already described in ex vivo studies using rat intestines.^51^ Nevertheless, the cell culture medium supplementation with glucose can influence the TEER values. Glucose is taken up by the cotransporter SGLT1, which simultaneously transport two Na^+^ ions into the cell.^52^ In the next step, the Na–K-ATPase transports three Na^+^ ions in the basolateral compartment or vascular system and three K^+^ ions into the cell.^52^ Such exchange and transport of ions may influence the TEER measurements. Interestingly, the effects of the apple extract fractions were different. The apple copigment fraction had no effect on the Papp index and the glucose transport. The apple polyphenols significantly increase glucose uptake, with no effects on sodium fluorescein transport. In the case of the apple extract, the effects were the other way around. Thus, the individual composition of the extracts had specific effects on the paracellular transport. It is known by previous studies that anthocyanins reduce the glucose uptake,^53–55^ but the anthocyanin content of apples is relatively low.^8^ Therefore, a reduction in glucose uptake was expected for the chokeberry extracts, which was not detected in a recent study. Apple extracts consist of chlorogenic acid, catechin, and quercetin glycosides.^8^ Many of these compounds specifically influence the tight junctions and intestinal permeability. In contrast to the recent results, Prpa and colleagues showed that the uptake of apple polyphenols such as epicatechin and quercetin reduced the glucose uptake. As more apple polyphenols were consumed, as lower was the glucose concentration in the urine.^56^ These differences in the influence of apple polyphenols on glucose uptake may be discussed by considering their combination with the sweeteners. Thus, in combination with saccharin, the polyphenols might increase the glucose uptake, which should be analyzed in more detail in the future.

Some studies already show that some bioactive compounds, in particular, polyphenols including phenolic acids and tannins, can influence the shape of the blood glucose curve and thus the uptake and transport of glucose. Further studies have shown that these compounds can lead to an altered pattern of intestinal glucose uptake possibly due to interactions between the compounds and enterocyte sugar transporters.^34,57,58^ To sum up, the recent results indicate a lower or nonaffected uptake of glucose by the sweeteners, while apple polyphenols in addition to the sweetener saccharin increase the glucose uptake. In future studies, the glucose concentration should be increased to 27 mM, which may show significant effects for more treatment combinations and is the upper limit of sugar in “sugar-free”-labeled beverages by EU regulation. Moreover, detailed analyses using the SGLT1-inhibitor phlorizin should be addressed to evaluate the affected cellular mechanisms by sweeteners and polyphenolic extracts.

### 4.4 qPCR

To reveal the impact and possible underlying mechanisms of the tested treatments, the transcription of related genes was analyzed. In the case of glucose uptake across the intestinal membrane, a fundamental distinction is made between sodium-dependent and sodium-independent glucose uptake (favoring uptake via SGLT1). As already described above, the treatment of the cells with the different sweeteners after a treatment time of 4 h did not result in a significant reduction in the expression of the glucose transporters GLUT1, GLUT2, and SGLT1 as well as on the proteolytic enzyme DPP4 compared to the control. Likewise, there was no significant change in the antioxidant enzymes CAT and SOD compared to the control or among the sweeteners (Figure 4 E,F). To summarize, the detected effects in glucose uptake did not rely on variations in the transcription of glucose transporters. Thus, tight junction proteins may be targeted by artificial sweeteners and fruit juice extracts.

Only a few studies have been conducted to date on the effects of sweeteners on glucose transporters. Some studies show that an effect on the transporter GLUT2, for example, as well as on SGLT1, depends on the sweetness of the respective sweeteners. In the studies by Xie et al.,^36^ there was a significant difference in the expression of SGLT1 between treatment with sucralose with a sweetening power of 36 and treatment with 20 mM glucose. At a glucose concentration of 60 mM, SGLT1 mRNA expression levels were comparable between the two treatments. For GLUT2, sucralose with a sweetness of 36 significantly increased mRNA levels at both 20 and 60 mM glucose concentrations. The expression levels of GLUT2 were significantly higher at the glucose concentration of 60 mM than at 20 mM. Another study indicated that when the glucose concentration was less than 25 mM, acesulfame K at a concentration of 10 mM had no significant effect on glucose absorption, but when the glucose concentration was above 25 mM, it had a promoting effect.^50^ This could be due to the different treatment times and the intensity of the sweetness of the artificial sweeteners studied. Other studies by Wright et al.^59^ and Wright et al.^60^ have shown that the classical sodium-glucose SGLT1 pathway and the diffusive apical GLUT2 pathway are the two major components of intestinal glucose absorption. In the studies by Xie et al. (Xie 2020), the mRNA expression level of GLUT2 was investigated after exposure to sucralose at a sweetness level of 36. Meanwhile, the mRNA expression of SGLT1 was strongly upregulated only at a glucose concentration of 20 mM, suggesting that GLUT2 has a higher capacity than SGLT1 for glucose.^61^ In addition, Xie and colleagues showed that artificial sweeteners were able to increase glucose transport via SGLT1 and GLUT2, depending on the intensity of sweetness.^36^ In future studies, the effects on glucose transporters as well as on tight junction proteins should be analyzed may including a longer treatment and Western blot analyses. Thus, changes can be detected on both the gene and protein level.

In the recent study, a concentration of 2 mM was tested for all sweeteners. 2 mM is the European maximum quantity for aspartame in aromatic beverages and fruit nectar (EU regulation 1333/2008) and below its ADI of 50 mg/kg/d. In the case of saccharin (ADI 5 mg/kg/d), cyclamate (ADI 1 mg/kg/d), and sucralose (ADI 5 mg/kg/d), the used concentration was above the ADI values and should be kept in mind by consideration of the results. There were no significant effects of cyclamate and sucralose on intestinal permeability. Thus, such effects seem to be unlikely under physiological conditions. The saccharin-induced increase in permeability possibly occurs after consumption of high levels, while the ADI was just slightly exceeded in the recent study and depended on the individual body weight of the consumer. In consideration of the compound-specific sweetness, comparable analyses could be interesting and should be performed in future studies. The sweetness of aspartame, saccharin, and acesulfame K are comparable (200-fold compared to glucose), while for cyclamate it is lower (30-fold) and for sucralose it is higher (600-fold). Therefore, the used concentrations of sucralose are nonrealistic for sugar-free beverages. However, no significant effects were seen for sucralose alone.

## 5 Conclusion

Artificially sweetened beverages supplemented with extracts from fruit juices could be a healthier alternative to sugar-sweetened beverages. According to our investigations, the copigment fraction from apples in combination with the sweeteners saccharin, aspartame, and sucralose could prevent or attenuate an increase in intestinal permeability. In combination with the sweeteners, this fraction may be promising in future in vivo and clinical trials for the treatment and prevention of chronic intestinal diseases. On the other hand, extracts of chokeberry in combination with the sweetener aspartame appear to increase intestinal permeability for several hours, while after 24 h, the permeability was recovered. Thus, these extracts can have a beneficial effect on health to increase the absorption of substances via the intestinal membrane. We were also able to show that when intestinal cells are treated with apple extract in combination with the sweetener saccharin, there is a higher permeability of sodium fluorescein through the intestinal barrier than with the other sweetener–fruit juice extract combinations. Glucose uptake was not affected by the sweeteners, but due to their combination with apple polyphenols, it significantly increased. In summary, depending on the aimed effects, a combination of artificial sweeteners with fruit juice extracts originating from apple or chokeberry could be beneficial ingredients in new healthier beverages.

## Supplementary Material



## Data Availability

All data supporting the findings of this study are available within the article and its Supporting Information.

## Notes

Primer sequences for qPCR analyses (PDF)

## References

[1] Stephens J. W., Brown K. E., Min T. (2020). Chronic kidney disease in type 2
diabetes: Implications for managing glycaemic control, cardiovascular
and renal risk. Diabetes Obes. Metabol..

[2] Malik V. S., Popkin B. M., Bray G. A., Després J.-P., Willett W. C., Hu F. B. (2010). Sugar-sweetened
beverages and risk
of metabolic syndrome and type 2 diabetes: a meta-analysis. Diabetes Care.

[3] Imamura F., O’Connor L., Ye Z., Mursu J., Hayashino Y., Bhupathiraju S. N., Forouhi N. G. (2015). Consumption of sugar sweetened beverages,
artificially sweetened beverages, and fruit juice and incidence of
type 2 diabetes: systematic review, meta-analysis, and estimation
of population attributable fraction. BMJ.

[4] Prinz P. (2019). The role of
dietary sugars in health: molecular composition or just calories?. Eur. J. Clin. Nutr..

[5] World Health Organization . Guideline: Sugars Intake for Adults and Children; World Health Organization, 2015. 25905159

[6] Ruxton C. H.
S., Myers M. (2021). Fruit Juices:
Are they helpful or harmful? An evidence
review. Nutrients.

[7] Nowak D., Gośliński M., Kłębukowska L. (2022). Antioxidant
and antimicrobial properties of selected fruit juices. Plant Foods Hum. Nutr..

[8] Köpsel M., Kostka T., Rodriguez-Werner M., Esatbeyoglu T. (2025). The influence
of fruit juice extracts on glucose intestinal transporters and antioxidant
genes in a Caco-2 and HT29-MTX co-culture cell system. Food Funct..

[9] Flavel M., Neoh J., Lim K. F. (2023). Dose-dependency of the glycemic response
to polyphenol-rich sugarcane extract (PRSE). Biologics.

[10] Agulló V., García-Viguera C., Domínguez-Perles R. (2022). The use of
alternative sweeteners (sucralose and stevia) in healthy soft-drink
beverages, enhances the bioavailability of polyphenols relative to
the classical caloric sucrose. Food Chem..

[11] Turner J. B., Kumar A., Koch C. A. (2016). The effects
of indoor and outdoor
temperature on metabolic rate and adipose tissue - the Mississippi
perspective on the obesity epidemic. Rev. Endocr.
Metab. Disord..

[12] Tandel K. R. (2011). Sugar substitutes:
Health controversy over perceived benefits. J. Pharmacol. Pharmacother..

[13] Weihrauch M. R., Diehl V. (2004). Artificial sweeteners—do they bear a carcinogenic risk?. Ann. Oncol..

[14] Whitehouse C. R., Boullata J., McCauley L. A. (2008). The potential toxicity of artificial
Sweeteners. AAOHN J..

[15] Bandyopadhyay R., Paul R., Basu A. K., Chakraborty P., Mitra S. (2013). Study of c reactive protein in type
2 diabetes and its relation with
various complications from Eastern India. J.
Appl. Pharm. Sci..

[16] Wiebe N., Padwal R., Field C., Marks S., Jacobs R., Tonelli M. (2011). A systematic review on the effect of sweeteners on
glycemic response and clinically relevant outcomes. BMC Med..

[17] Greenwood D. C., Threapleton D. E., Evans C. E. L., Cleghorn C. L., Nykjaer C., Woodhead C., Burley V. J. (2014). Association between sugar-sweetened
and artificially sweetened soft drinks and type 2 diabetes: systematic
review and dose-response meta-analysis of prospective studies. Br. J. Nutr..

[18] Bruyère O., Ahmed S. H., Atlan C., Belegaud J., Bortolotti M., Canivenc-Lavier M. C., Charrière S., Girardet J.-P., Houdart S., Kalonji E., Nadaud P., Rajas F., Slama G., Margaritis I. (2015). Review of
the nutritional benefits and risks related
to intense sweeteners. Arch. Public Health.

[19] Ray S., Palui R. (2025). Artificial sweeteners: Benefits, risks and controversy. Apollo Med..

[20] Gougeon R., Spidel M., Lee K., Field C. J. (2004). Canadian
diabetes
association national nutrition committee technical review: non-nutritive
intense sweeteners in diabetes management. Can.
J. Diabetes.

[21] Brown R. J., de Banate M. A., Rother K. I. (2010). Artificial sweeteners: a systematic
review of metabolic effects in youth. Int. J.
Pediatr. Obes..

[22] Conz A., Salmona M., Diomede L. (2023). Effect of
non-nutritive sweeteners
on the gut microbiota. Nutrients.

[23] Lobach A. R., Roberts A., Rowland I. R. (2019). Assessing the in vivo data on low/no-calorie
sweeteners and the gut microbiota. Food Chem.
Toxicol..

[24] Suez J., Korem T., Zeevi D., Zilberman-Schapira G., Thaiss C. A., Maza O., Israeli D., Zmora N., Gilad S., Weinberger A., Kuperman Y., Harmelin A., Kolodkin-Gal I., Shapiro H., Halpern Z., Segal E., Elinav E. (2014). Artificial
sweeteners induce glucose intolerance by
altering the gut microbiota. Nature.

[25] Nettleton J. E., Reimer R. A., Shearer J. (2016). Reshaping
the gut microbiota: Impact
of low calorie sweeteners and the link to insulin resistance?. Physiol. Behav..

[26] König J., Wells J., Cani P. D., García-Ródenas C. L., MacDonald T., Mercenier A., Whyte J., Troost F., Brummer R. J. (2016). Human intestinal barrier function in health and disease. Clin. Transl. Gastroenterol..

[27] Santos P. S., Caria C. R. P., Gotardo E. M. F., Ribeiro M. L., Pedrazzoli J., Gambero A. (2018). Artificial sweetener saccharin disrupts intestinal
epithelial cells’ barrier function in vitro. Food Funct..

[28] Kostka T., Ostberg-Potthoff J. J., Stärke J., Guigas C., Matsugo S., Mirčeski V., Stojanov L., Veličkovska S. K., Winterhalter P., Esatbeyoglu T. (2022). Bioactive Phenolic Compounds from
Lingonberry (Vaccinium vitis-idaea L.): Extraction, chemical characterization,
fractionation and cellular antioxidant activity. Antioxidants.

[29] Niesen S., Göttel C., Becker H., Bakuradze T., Winterhalter P., Richling E. (2022). Fractionation of extracts from black
chokeberry, cranberry, and pomegranate to identify compounds that
influence lipid metabolism. Foods.

[30] Juadjur A., Winterhalter P. (2012). Development of a novel adsorptive membrane chromatographic
method for the fractionation of polyphenols from bilberry. J. Agric. Food Chem..

[31] Ferraretto A., Bottani M., de Luca P., Cornaghi L., Arnaboldi F., Maggioni M., Fiorilli A., Donetti E. (2018). Morphofunctional properties
of a differentiated Caco2/HT-29 co-culture as *an in vitro* model of human intestinal epithelium. Biosci.
Rep..

[32] Misawa N., Honda S. (2023). Increased sodium fluorescein
transport by corticosteroids is inhibited
by a LAT-1 specific inhibitor in retinal pigment epithelial cells *in vitro*. Sci. Rep..

[33] Castro-Acosta M. L., Stone S. G., Mok J. E., Mhajan R. K., Fu C.-I., Lenihan-Geels G. N., Corpe C. P., Hall W. L. (2017). Apple and
blackcurrant
polyphenol-rich drinks decrease postprandial glucose, insulin and
incretin response to a high-carbohydrate meal in healthy men and women. J. Nutr. Biochem..

[34] Johnston K., Sharp P., Clifford M., Morgan L. (2005). Dietary polyphenols
decrease glucose uptake by human intestinal Caco-2 cells. FEBS Lett..

[35] van
Eyk A. D. (2015). The effect of five artificial sweeteners on Caco-2, HT-29 and HEK-293
cells. Drug Chem. Toxicol..

[36] Xie N., Huang X., Yang C., Dai M., Cai L., Deng S., Hardiman P. J., Zhou J. (2020). Artificial
sweeteners
affect the glucose transport rate in the Caco-2/NCI-H716 co-culture
model. J. Sci. Food Agric..

[37] Zheng Z., Xiao Y., Ma L., Lyu W., Peng H., Wang X., Ren Y., Li J. (2022). Low Dose of Sucralose
Alter Gut Microbiome in Mice. Front. Nutr..

[38] Bian X., Chi L., Gao B., Tu P., Ru H., Lu K. (2017). Gut Microbiome
Response to Sucralose and Its Potential Role in Inducing Liver Inflammation
in Mice. Front. Physiol..

[39] Shil A., Olusanya O., Ghufoor Z., Forson B., Marks J., Chichger H. (2020). Artificial sweeteners
disrupt tight junctions and barrier
function in the intestinal epithelium through activation of the sweet
taste receptor, T1R3. Nutrients.

[40] Margolskee R. F., Dyer J., Kokrashvili Z., Salmon K. S., Ilegems E., Daly K., Maillet E. L., Ninomiya Y., Mosinger B., Shirazi-Beechey S. P. (2007). T1R3 and gustducin in gut sense sugars to regulate
expression of Na+-glucose cotransporter 1. Proc.
Natl. Acad. Sci. U.S.A..

[41] Lamson N. G., Fein K. C., Gleeson J. P., Newby A. N., Xian S., Cochran K., Chaudhary N., Melamed J. R., Ball R. L., Suri K., Ahuja V., Zhang A., Berger A., Kolodieznyi D., Schmidt B. F., Silva G. L., Whitehead K. A. (2022). The strawberry-derived
permeation enhancer pelargonidin enables oral protein delivery. Proc. Natl. Acad. Sci. U.S.A..

[42] Rowland I., Gibson G., Heinken A., Scott K., Swann J., Thiele I., Tuohy K. (2018). Gut microbiota functions:
metabolism
of nutrients and other food components. Eur.
J. Nutr..

[43] Mercado J., Valenzano M. C., Jeffers C., Sedlak J., Cugliari M. K., Papanikolaou E., Clouse J., Miao J., Wertan N. E., Mullin J. M. (2013). Enhancement
of tight junctional barrier function by
micronutrients: compound-specific effects on permeability and claudin
composition. PLoS One.

[44] Carrasco-Pozo C., Morales P., Gotteland M. (2013). Polyphenols
protect the epithelial
barrier function of Caco-2 cells exposed to indomethacin through the
modulation of occludin and zonula occludens-1 expression. J. Agric. Food Chem..

[45] Sharma S., Tripathi P., Sharma J., Dixit A. (2020). Flavonoids modulate
tight junction barrier functions in hyperglycemic human intestinal
Caco-2 cells. Nutrition.

[46] Srinivasan B., Kolli A. R., Esch M. B., Abaci H. E., Shuler M. L., Hickman J. J. (2015). TEER measurement techniques for *in vitro* barrier model systems. J. Lab. Autom..

[47] Barberis A., Garbetta A., Cardinali A., Bazzu G., D’Antuono I., Rocchitta G., Fadda A., Linsalata V., D’Hallewin G., Serra P. A., Minervini F. (2017). Real-time
monitoring of glucose and phenols intestinal absorption through an
integrated Caco-2TC7cells/biosensors telemetric device: Hypoglycemic
effect of fruit phytochemicals. Biosens. Bioelectron..

[48] Kausalya P. J., Amasheh S., Günzel D., Wurps H., Müller D., Fromm M., Hunziker W. (2006). Disease-associated
mutations affect
intracellular traffic and paracellular Mg2+ transport function of
Claudin-16. J. Clin. Invest..

[49] Sandle G. I., Rajendran V. M. (2025). Ion transport and epithelial barrier dysfunction in
experimental models of ulcerative colitis. Am.
J. Physiol. Gastrointest. Liver Physiol..

[50] Zheng Y., Sarr M. G. (2013). Effect of the artificial sweetener,
acesulfame potassium,
a sweet taste receptor agonist, on glucose uptake in small intestinal
cell lines. J. Gastrointest. Surg..

[51] Bæch-Laursen C., Kuhre R. E., Modvig I. M., Veedfald S., Holst J. J. (2025). Glucose
absorption by isolated, vascularly perfused rat intestine: A significant
paracellular contribution augmented by SGLT1 inhibition. Acta Physiol..

[52] Koepsell H. (2020). Glucose transporters
in the small intestine in health and disease. Pflügers Archiv.

[53] Lee T.-W., Song Y.-B., Kim C. Y., Lee J. H., Lee B.-H. (2025). Regulation
of glucose uptake level by black corn-derived anthocyanins at the
small intestinal α-Glucosidases and different types of glucose
transporters. J. Agric. Food Chem..

[54] Zhang P.-W., Chen F.-X., Li D., Ling W.-H., Guo H.-H. (2015). A CONSORT-compliant,
randomized, double-blind, placebo-controlled pilot trial of purified
anthocyanin in patients with nonalcoholic fatty liver disease. Medicine.

[55] Alzaid F., Cheung H.-M., Preedy V. R., Sharp P. A. (2013). Regulation of glucose
transporter expression in human intestinal Caco-2 cells following
exposure to an anthocyanin-rich berry extract. PLoS One.

[56] Prpa E. J., Corpe C. P., Atkinson B., Blackstone B., Leftley E. S., Parekh P., Philo M., Kroon P. A., Hall W. L. (2020). Apple polyphenol-rich drinks dose-dependently
decrease
early-phase postprandial glucose concentrations following a high-carbohydrate
meal: a randomized controlled trial in healthy adults and in vitro
studies. J. Nutr. Biochem..

[57] Manzano S., Williamson G. (2010). Polyphenols and phenolic acids from strawberry and
apple decrease glucose uptake and transport by human intestinal Caco-2
cells. Mol. Nutr. Food Res..

[58] Kwon O., Eck P., Chen S., Corpe C. P., Lee J.-H., Kruhlak M., Levine M. (2007). Inhibition of the intestinal
glucose transporter GLUT2
by flavonoids. FASEB J..

[59] Wright E. M., Loo D. D. F., Hirayama B. A. (2011). Biology of human
sodium glucose transporters. Physiol. Rev..

[60] Wright E. M., Ghezzi C., Loo D. D. F. (2017). Novel
and unexpected functions of
SGLTs. Physiology.

[61] Kellett G. L., Helliwell P. A. (2000). The diffusive
component of intestinal glucose absorption
is mediated by the glucose-induced recruitment of GLUT2 to the brush-border
membrane. Biochem. J..

