# Supplementary material for: In Vitro Testing of Artificial Sweeteners with Apple and Chokeberry Fruit Juice Extracts for Intestinal Permeability and Glucose Uptake
Source: ACS Omega. 2026 Mar 31;11(14):21577–89. doi: 10.1021/acsomega.5c06005 (PMC13084496; doi:10.1021/acsomega.5c06005)
Supplement: Supplementary file 1 [file ao5c06005_si_001.pdf]

***In vitro* testing of artificial sweeteners with apple and chokeberry fruit juice extracts for intestinal permeability and glucose uptake**

Magdalena Köpsel[1], Tina Kostka[2], Tuba Esatbeyoglu[1]*

[1] Department of Molecular Food Chemistry and Food Development, Institute of Food Science and Human Nutrition, Gottfried Wilhelm Leibniz Universität Hannover, Am Kleinen Felde 30, 30167 Hannover; koepsel@lw.uni-hannover.de; esatbeyoglu@lw.uni-hannover.de

[2] Division of Food Chemistry and Toxicology, Department of Chemistry, RPTU Kaiserslautern-Landau, Erwin-Schrödinger-Strasse 52, 67663 Kaiserslautern, Germany; kostka@rhrk.uni-kl.de

Corresponding author: Prof. Dr. Tuba Esatbeyoglu, email: esatbeyoglu@lw.uni-hannover.de

Supplementary data

Supplementary Table S1 Primer sequences for genes analyzed by qPCR and qPCR validation data.

Gene name	Forward 5'->3'	Reverse 5'->3'	slope	R^2	efficiency [%]
GAPDH	AGCCACATCGC TCAGACAC	GCCCAATACG ACCAAATCC	-3.276	1	101.940
18S	ATCAACTTTCG ATGGTAGTCG	TCCTTGGATG TGGTAGCCG	-3.299	1	100.949
GLUT1	TCCTGAGACTA AAGGCCGGA	TCACACTTGG GAATCAGCCC	-3.210	0.999	104.879
GLUT2	GACAGAAGATA AGGTCACTGGG	CCAGTGGAAC ACCCAAAACA	-3.269	0.998	102.253
SGLT	GTCGGACTGTG GGCTATGTT	GCAAAGAGGG AGGCTCCAAT	-3.189	0.999	105.863
DPP4	CACCTGGGAAG TCATCGGG	AGATTCCTTC CTCCTGGCAT TC	-3.175	0.998	106.510
CAT	GACCATTGCAT CATTGGCCG	CAATTACACC ACAAGCCAAA CG	-3.311	0.994	100.450
SOD	ACTTCTGGAGC CTACGTCCT	TCTGGAATCC CCCGATCACT	-3.197	0.999	105.487
